# “Handing out non-prescribed antibiotics is storing up trouble for the next generation!” Unpacking multistakeholder views of drivers and potential solutions in Ethiopia

**DOI:** 10.1186/s12913-023-09819-4

**Published:** 2023-08-07

**Authors:** Sewunet Admasu Belachew, Lisa Hall, Linda A Selvey

**Affiliations:** 1 School of Public Health, The University of Queensland, 288 Herston Rd, Herston, Qld 4006 Australia; 2 School of Pharmacy, Faculty of Medicine and Health Sciences, University of Gondar, Gondar, Ethiopia

**Keywords:** Driver, Ethiopia, Multi-stakeholder, Non-prescribed antibiotics, Perspectives, Pharmacy professional

## Abstract

**Background:**

Antibiotic resistance is a global health crisis, with inappropriate antibiotic use often being linked to non-prescribed antibiotic dispensing practices. This study aimed to examine the perspectives of multiple stakeholders on the drivers and potential solutions for non-prescribed antibiotic dispensing in Ethiopian community drug retail outlets (CDROs). Despite the prescription only use policies, the practice remains prevalent in Ethiopia. Many factors are thought to contribute to this issue, although little research is available for non-urban settings.

**Methods:**

A phenomenological qualitative study was conducted. Pharmacy professionals (owners or employees) working in non-urban towns CDROs were selected through a simulated client study, which identified CDROs that had dispensed antibiotics without a prescription. Some high-level decision makers in the Ethiopian health system were also purposively selected. Interviews were conducted in-person and over the phone or via Zoom. The interview data were transcribed verbatim, translated to English, and thematically analysed. NVivo 12 software was used to assist with coding.

**Results:**

CDRO pharmacy professionals (n = 18) and five decision makers were interviewed. Most professionals (61%) were pharmacists working in drug stores, with one to 11 years of work experience. Several contributing factors were identified at the level of patients, CDRO staff, and the healthcare system. These included economic interests, inadequate knowledge and inappropriate attitudes about antibiotic use or supply, and issues within the healthcare system included inaccessibility and insufficient capacity, absence of or a weak enforcement of prescription-only regulations or service supervision. Additionally, patient-related factors included a lack of knowledge and inappropriate attitudes about antibiotics use and their supply, previous successful treatment experience and a culture of seeking out antibiotics.

**Conclusions:**

A complex set of modifiable factors related to patients, CDRO staff and healthcare system were identified that contribute to the non-prescribed supply of antibiotics. Due to this complexity, a single solution will not resolve the issues. Therefore, a range of multifaceted solutions have been suggested, including stricter regulation, increasing availability and accessibility of healthcare services, collaboration, and local consensus-building among CDROs, regular training for CDRO staff, and using community social events to educate the public about responsible use of antibiotics.

**Supplementary Information:**

The online version contains supplementary material available at 10.1186/s12913-023-09819-4.

## Introduction

Antibiotics are widely used medicines, with a significant increase in consumption globally after the year 2000 [1]. However, the efficacy of antibiotics in preventing and treating common infections is being hindered by the prevalent issue of antibiotic resistance (ABR). It is estimated that ABR causes 700,000 deaths worldwide each year and results in significant economic loss [2]. If left unchecked, this number could rise to 10 million deaths by 2050 [2, 3]. The relationship between antibiotic use and the development of ABR is well documented [4–8]. Although ABR can occur naturally, it is largely believed to be caused by overuse or misuse of antibiotics [9–13].

Self-medication with antibiotics, which are often obtained from community drug retail outlets (CDROs) in low- and middle-income countries (LMICs) [14–16], has been identified as a leading contributor to the overuse of antibiotics including Ethiopia [17–21]. This type of misuse or overuse has negative consequences including bacterial resistance, wastage of resources, and adverse drug outcomes [22, 23]. Despite laws prohibiting the sale of antibiotics without prescription, this practice remains common in many LMICs [9, 17, 24]. Factors that contribute to this include the accessibility of CDROs, pressure from customers, financial incentives for staff, lack of enforcement of regulations and the knowledge and attitudes of CDRO staff and patients [25, 26].

In Ethiopia dispensing of antibiotics without prescription is contrary to the pharmacy professionals’ professional ethics and Ethiopian legislation requires prescription-only sale of antibiotics. The scale of antibiotic dispensing without prescription in Ethiopia is believed to be greater in the rural areas, where there may be challenges to accessing healthcare services. Recent studies conducted in non-urban towns of the Amhara region CDROs in Ethiopia have revealed the high rate of sale of antibiotics without prescription from CDROs [18, 27]. This highlights the need for further research involving stakeholders to better understand and deepen our knowledge of the drivers for the reported high rate of non-prescribed supply of antibiotics and to inform evidence-based interventions to address it.

We aimed to explore the views of pharmacy professionals working in non-urban towns CDROs and high-level decision makers from the health system in Ethiopia on the drivers of and potential solutions to non-prescribed antibiotic dispensing practice.

## Methods

### Study design

This qualitative study, using a phenomenological approach, was conducted through semi-structured interviews with CDRO staff and high-level decision makers. A phenomenological study design is one of the most commonly used methodologies in qualitative research [28]. This study design aims to understand the lived experiences or thoughts and viewpoints of study participants on a range of issues related to this phenomenon, including the reasons for selling antibiotics without prescription and the potential solutions to address it. It delivers information about unique individual experiences or perspectives. Specifically, this study employed a descriptive phenomenological design, describing the participants experiences and/or perspectives, without imposing any preconceived theories. Data collection in phenomenological research involves long and intensive, semi-structured or unstructured interviews. It heavily relies on interviews, requiring researchers to conduct several interview sessions [28]. We have selected a phenomenological design for our study on the non-prescribed sale of antibiotics and potential solutions to address it. This is because our goal is to explore the lived experiences or thoughts and perspectives of pharmacy professionals and key stakeholders related to this issue. This approach allows the capture of the diversity and complexity of the participants’ experiences and viewpoints, which can inform the development of more effective interventions and policies [28].

Furthermore, the study adhered to the Consolidated Criteria for Reporting Qualitative Studies (COREQ) to report the views of CDRO staff working in non-urban towns CDROs and high-level decision makers in the health system of Ethiopia on the reasons for the reported and observed higher prevalence of non-prescribed antibiotics dispensing practice in Ethiopia, and possible strategies to counteract the practice [29].

### Sample size determination and sampling technique/procedure

This study builds up on two previous quantitative studies (a survey of knowledge, attitudes and practices, and a simulated client study) conducted in non-urban towns of the Amhara region, Ethiopia [18, 27] to examine the dispensing of non-prescribed antibiotics. To achieve the study’s goal, interviews were conducted with pharmacy professionals working in non-urban towns CDROs where antibiotics were found to have been dispensed without prescription in the previous studies [18, 27]; and high-level decision makers involved in medicine regulation or the health system of Ethiopia who were identified as key stakeholders in fighting antimicrobial resistance (AMR). A maximum variation sampling technique was used to gather data from a diverse group of pharmacy professionals from different types of CDROs, locations, and demographics. The CDROs were identified in the previous studies [18, 27] and potential interviewees were approached and invited to participate. The final sample size of pharmacy professionals was determined using the concept of data saturation. After interviewing 15 pharmacy professionals the same themes were consistently being identified and we stopped findings new codes or themes or ideas. This indicated that the data saturation had been achieved. However, three additional participants were interviewed to ensure saturation. On the other hand, for the high-level decision makers interview, a pre-determined number of participants were selected from relevant health sectors and institutions in Ethiopia that were identified by the government as key stakeholders in the fight against AMR (including the Ethiopian Ministry of Health, Ethiopian Food and Drug Authority, Ethiopian Pharmaceuticals Association, and Ethiopian Pharmaceuticals Supply Agency). Potential participants from these sectors were approached, with a preference given to those who held current roles or positions in the selected sectors or institutions whenever possible. The reason for choosing these stakeholders is because they are among the most responsible for making decisions that directly affect the implementation of policies and guidelines related to AMR. Resources available for the study did not permit us to interview all stakeholders involved in the fight against AMR in Ethiopia.

### Data collection tool and approach

A semi-structured interview guide was used to conduct the interviews. The guide was developed by the researchers based on the study’s objectives. The guide was created by considering the potential issues that could result in the dispensing of non-prescribed antibiotics in Ethiopia, drawing upon the previously published studies [17, 18, 25, 27], and organising those concepts to form the structure of the guide. The guide was initially developed in English and then translated into Amharic, the local language commonly used, as all the interviews were conducted in Amharic. The interview questions were divided into three sections, each with prompts (Additional file 1). The first section focused on personal and professional information, the second section covered pharmacy professionals’ views on the current antibiotic dispensing practices from CDROs, including reasons for the non-prescribed provision of antibiotics and strategies for improvement. The phenomenon of interest was also further explored through the last section that focused on antibiotic dispensing regulation or the use of prescription-only antibiotics. A shorter version of the guide was used for the high-level decision makers’ interviews. The semi-structured interview guide included pre-prepared questions and topics and also allowed for follow-up questions based on the participant’s response. This approach ensured that the interviewers stayed on the topic of interest while also leaving a room for any additional ideas that arose during the interview.

The researcher (SAB) recruited two interviewers with prior experience in collecting qualitative data. To ensure consistency in data collection, one interviewer conducted all interviews with the pharmacy professionals, while the other interviewed the decision makers. Before the interviews began, the researcher (SAB) provided training and orientation to the interviewers. The training included an overview of the interview guide and potential prompts, techniques for approaching CDRO staff and effective listening, obtaining consent, and audio-recording. Before collecting data, the interviewers conducted mock interviews with each other, and with the researcher (SAB) to test the interview guide, identify any potential issues with the questions or prompts, and the interview process. It was also an opportunity for the interviewers to practice/refresh their interviewing skills and receive feedback from the researchers. Additionally, to ensure the quality of the interview, the interviewers sent the first few recordings to the researcher (SAB) after each interview and received feedback before conducting the next interview. Feedback was provided on aspects such as recording sound quality, the introduction of the interview, and suggestions for probing points to consider.

The pharmacy professionals were interviewed face-to-face at their CDROs or at convenient locations. The researcher (SAB) identified potential interviewees with the help of the field supervisor of the project. The interviewers then approached potential participants and verbally briefed them before providing them with written participant information sheet about the study and obtaining written informed consent for participation and audio-recording. The decision makers’ interviews were conducted via telephone and one decision maker was interviewed by the researcher (SAB) via Zoom (Zoom Video Communications, Inc., San Jose, California, USA). All interviews were conducted in Amharic language using the interview guide and were audio-recorded. The pharmacy professionals’ interviews lasted an average of 40 min, ranging from 28 to 60 min, and decision makers’ interviews lasted an average of 28 min.

### Ethical considerations

The study was approved by the University of Queensland Human Research Ethics Committee (approval number 2,020,002,195), Australia and University of Gondar Institutional Ethical Review Board, Ethiopia (approval number V/P/RCS/05/412/2020). All methods were carried out in accordance with the relevant guidelines and regulations [29, 30].

### Quality assurance and data analysis

All the audio recordings were transcribed verbatim and two of them translated to English by one of the researchers (SAB). To ensure accuracy and avoid uncertainties, the researcher (SAB) listened to the audio recordings multiple times and reviewed the transcripts and translations. To verify the quality and consistency, the transcriptions and translation of the two audio-recordings were cross checked by an invited pharmacist. The de-identified data were imported into, and coding was performed using NVivo software version 12 (QSR International Pty Ltd., Doncaster, Victoria, Australia).

We followed the six-phase method of thematic analysis developed by the Braun and Clark (2006) to analyse the data [31, 32]. The process involved the following steps: (i) familiarisation, which required becoming familiar with the data by multiple times reading while taking initial notes, focusing on patterns that occur, (ii) coding, which involved highlighting specific parts of the text such as phrases or sentences, and collapsing the data into a shortened labels or codes to describe their content; (iii) generating themes through examining codes created, identifying patterns among them to collapse into themes. These themes were typically broader than codes, as several codes can be combined into a single theme; (iv) reviewing themes, which involved evaluating the themes and ensuring that they accurately represented the data. At this stage, themes were split up, combined, discarded, or new themes were created to make them more useful and accurate; (v) defining and naming themes, which included articulating precisely what each theme meant and how it helped to understand the data. This also involved formulating a concise and easily understandable name for each theme; (vi) write-up, which entailed writing the analysis of the data, as well as an introduction that included the research question, aims and approach.

One of the researchers (SAB) performed line-by-line coding of the Amharic transcripts using Nvivo software and identified preliminary themes. The preliminary themes were then reviewed, defined, and named through discussion and consensus among all researchers (SAB, LH, LAS).

#### Definitions of terms

##### CDROs

refers to medicine shops, including pharmacies, drug stores and rural drug vendors [33].

##### Pharmacies

are medicine shops supervised by professionally registered pharmacists (with a degree level) who dispense medicines with or without prescription and compound medicines as directed by a physician. Compared to drug stores and rural drug vendors, a wider range of medicines are allowed to be stocked in Pharmacies [33].

##### Drug stores

are medicine shops supervised by professionally registered pharmacists (with a degree level) or druggists (with a diploma level). The scope of service provided in drug stores is less than that provided in Pharmacies, and a smaller range of medicines are also allowed to be stocked in drug stores compared to Pharmacies [33].

##### Rural drug vendors

are also medicine shops supervised by professionally registered druggists (with a diploma level). The scope of service provided by rural drug vendors is minimal compared to that provided in drug stores and Pharmacies. The range of medicines allowed to be stocked in these medicine shops is much less than in drug stores and Pharmacies. These medicine shops are commonly found in rural areas [33].

##### Inappropriate attitudes about antibiotic use or their supply

refers to attitudes or behaviours that contribute to inappropriate use or overuse of antibiotics, such as demanding or providing antibiotics when they are not needed due to a belief that antibiotics are beneficial in most instances, believing antibiotics as over-the counter medicines or other commodities and self-medication with antibiotic without medical supervision.

## Results

### Participants

Eighteen pharmacy professionals working in CDROs located in non-urban towns and five high-level decision makers in the health system of Ethiopia participated in the interview. The average age of the pharmacy professionals was 29 (± 4) years and their work experience varied from one to 11 years. The majority of pharmacy professionals, 61%, were pharmacists and worked at drug stores (Table 1).

**Table 1:** Pharmacy professionals’ demographic information

Characteristics	Frequency (%)
**Gender**	
Male	12 (67)
Female	6 (33)
**Age in years**	
20–29	8 (44)
30–39	10 (56)
**CDROs working in**	
Pharmacy	6 (33)
Drug store	11 (61)
Rural drug vendor	1 (6)
**Year of experience**	
1–4	13 (72)
>5	5 (28)
**Education level**	
Pharmacist	11 (61)
Druggist	7 (39)
**Employment status**	
Owner	11 (61)
Employee	7 (39)

### Themes

Three themes and seven sub-themes were identified after analysing the drivers behind the supply of antibiotics without prescription from CDROs. The three themes include CDRO staff related factors including economic interests; health system-related factors including inaccessibility and inadequate capacity of healthcare facility services; and patient-related factors including lack of knowledge and inappropriate attitudes towards antibiotic use or supply.

**Fig. 1:**
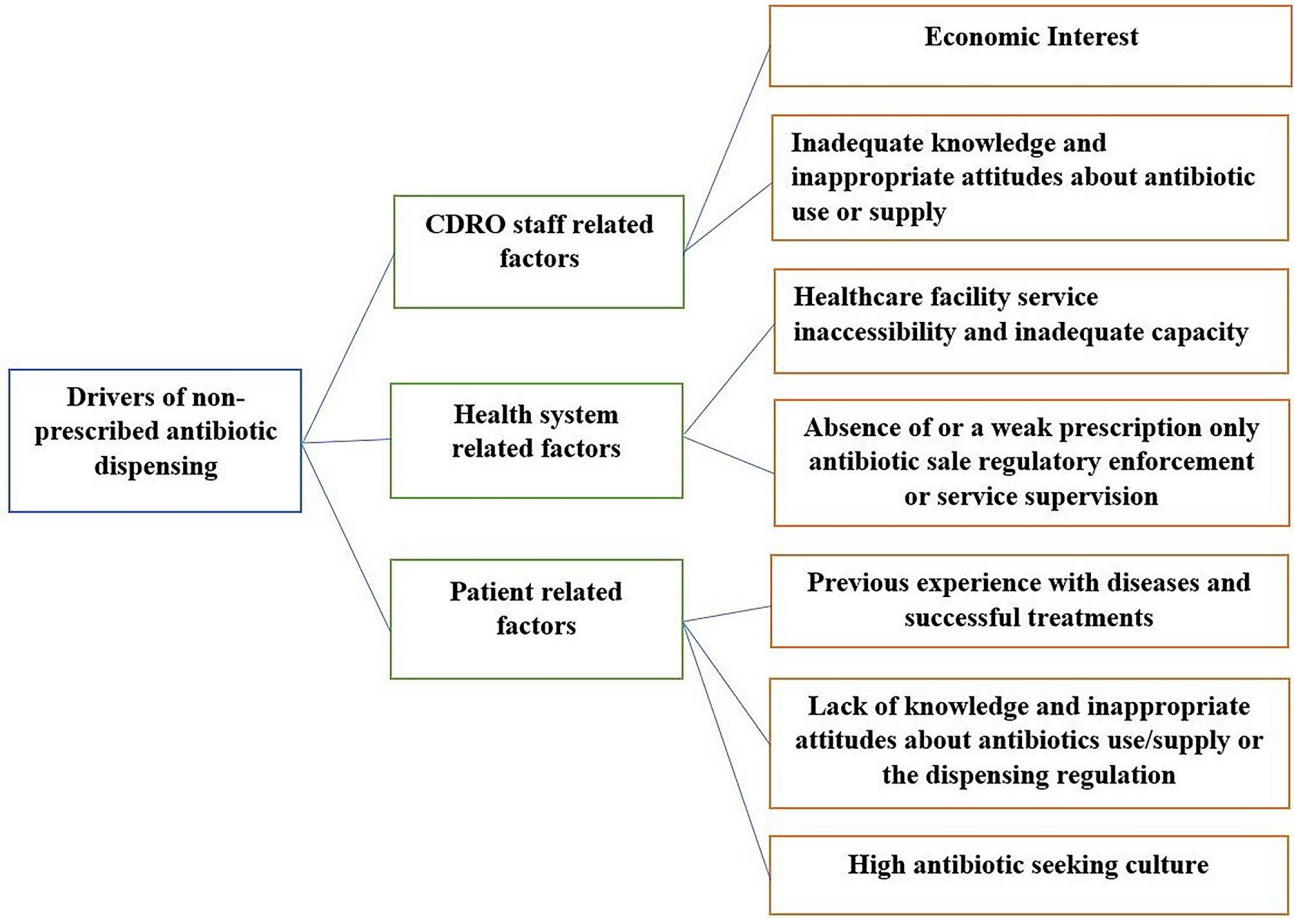
Themes and sub-themes

The primary themes and sub-themes are illustrated in Fig. 1, and further discussed in the following text.

### CDRO staff related factors

#### Economic interest: incentives relating to CDRO profits

Most pharmacy professionals and all the decision makers said that a desire for high profit has played a critical role in driving the dispensing of non-prescription antibiotics. The majority of participants emphasised that compliance with dispensing regulations and refusing to provide non-prescribed antibiotics would greatly impact the income of the CDRO, which in turn, would negatively affect the CDRO’s ability to cover routine expenses such as rent, taxes, and employees’ salaries. Many of the decision makers felt that the profit-driven aspect of CDRO services greatly compromised ethical pharmacy practices.

*“I believe dispensing antibiotics according to the regulations will affect the pharmacy income. As a result, the pharmacy could not afford to pay rental fees of the premises, tax, and employees’ salary. This eventually leads the pharmacy to be marginalised from the market”* (P11**)**

*“Fundamentally, pharmacy by nature has a business component. Sometimes, instead of refusing to dispense prescription only medicines without prescription, providing them would benefit the pharmacy in terms of avoiding financial struggle to pay rent, employees’ salary, and other expenses”* (DM 4)

Approximately half of the pharmacy professionals indicated that restricting the sale of antibiotics to prescription-only could lead to the loss of customers in competitive markets, which would directly affect CDRO income. The pharmacy professionals reported that they retain customers by accommodating non-prescribed antibiotic dispensing to make them happy, despite knowing it is against regulations. Some participants also reflected CDROs’ fear of being pushed out of the pharmacy business if they continue to deny non-prescribed antibiotics, as losing customers is a commonly reported consequence of refusal.

*“If I commit to practice according to the regulations, patients never accept it rather they decide to stop coming to my CDRO again and even tag me as a very conservative and unsupportive person. So, I cannot refuse selling non-prescribed antibiotics because I do not want to lose my customers, they are my sole source of income”* (P8)

Most pharmacy professionals and a single decision maker noted that business competition among rival CDROs is a major factor driving the supply of antibiotics without prescription. Many pharmacy professionals stated that if a CDRO declines to dispense antibiotics, clients will often go to a neighbouring CDRO and be successful in obtaining them. They explained that CDROs compete to attract more customers in order to maintain financial benefits. Most pharmacy professionals said that this lack of uniformity in dispensing practices substantially affects the income of those who adhere to the prescription-only antibiotic dispensing regulation. As a result, many CDROs choose to always comply with clients’ requests for antibiotics in order to survive in such a competitive environment.

*“There are also instances were clients straightforward mentioned that if you do not give me, I can easily get it from other CDROs. At this occasion, I always decide to provide them with what they have requested with no pre-condition because; they are my customers and do not want to handover my customers to other CDROs and lose my business”* (P15**)**

Many pharmacy professionals and one decision maker stated that CDRO owners pressure them to act in the best interest of the CDRO, which includes dispensing non-prescription antibiotics regardless of regulations. This pressure from owners or employers reflects their primary goal of maximising revenues.

*“The employers only care about their financial benefit that they do not want to see their income go lower than they expected. Thus, they insist that we sell antibiotics disregarding the regulation since their primary goal is maximising the CDRO revenue”* (P2)

*“The owners’ pressure significantly impacts CDRO staff attitude and practice, and in the long run, the CDRO staff become loyal executers of the owners’ interests whether it is appropriate or not”* (DM3)

### Inadequate knowledge and inappropriate attitudes about antibiotics use or supply

The majority of pharmacy professionals had inadequate awareness and inappropriate attitudes towards antibiotic use or provision, which contributes to the supply of antibiotics without prescription. Some of the pharmacy professionals stated that it is acceptable to provide antibiotics for treating conditions they deemed mild, such as fever. Additionally, the majority of them believed that providing non-prescription antibiotics to patients is not problematic as long as the patient is an adult.

Some pharmacy professionals believed that they have the necessary expertise to evaluate and prescribe antibiotics for certain conditions, such as upper respiratory tract infections. Their confidence in diagnosing and treating these conditions was an important reason for supplying antibiotics without prescription. Many of them believed that patients do not recover without antibiotics and thus using antibiotics in most cases is beneficial, particularly using broad-spectrum antibiotics such as amoxicillin.

The inappropriate attitudes of pharmacy professionals towards antibiotic dispensing regulations were also discussed. Some of them believed that it is acceptable to provide non-prescribed antibiotics to a family member, despite being aware of the prescription-only use rule. Some also believed that not all antibiotics are restricted to prescription-only use, and that some antibiotics such as amoxicillin do not require a prescription. Some pharmacy professionals treated antibiotics as over-the-counter medicines. In addition, pharmacy professionals were not aware of the consequences or repercussions of selling antibiotics without prescription.

The majority of decision makers highlighted that CDRO staff’s lack of knowledge about antibiotic use or the dispensing regulations is a contributing factor to their practice of dispensing antibiotics without prescription.

*“When I assume that the case is mild, I usually decide to provide antibiotics without prescription. However, when I feel that the case is severe or chronic, I refer the clients to healthcare facilities”* (P10)

*“There are instances where the CDRO staff think of antibiotics as if they are over-the-counter medicines”* (DM5)

Some pharmacy professionals believed that providing non-prescribed antibiotics is a reasonable decision based on patients’ individual circumstances such as financial limitations for consulting experts/specialists or that the patient’s condition is believed to be urgent or acute requiring immediate treatment.

*“When a child or adult comes with acute diarrhoea; by believing that the patient may go tomorrow to clinic or the clinic might be far from their home that until they visit or reach the clinic, I will not let the patients go without medication and increase risk of death, rather I will give them antibiotics for immediate relief although I know that antibiotics cannot be used as a pain killer”* (P1)

### Health system related factors

#### Healthcare facility service inaccessibility and inadequate capacity

Most of the pharmacy professionals disclosed that a lack of healthcare facilities in nearby areas or poor quality or satisfactory services at available facilities, such as long waiting times, unfriendly staff, and absence of or insufficient diagnostics or laboratory service, contribute to patients seeking non-prescription antibiotics from CDROs. All decision makers concurred that limited access to healthcare facilities is a major factor driving community members to rely on CDROs for consultations or medicine, including antibiotics without a prescription.

*“As the healthcare facilities are far from here and the facilities are usually overcrowded with a high number of patients causing a very prolonged waiting time to get the service, the patients directly come to our CDRO to get around of such inconveniences”* (P10)

*“Patients do not need the overcrowded services of the healthcare facilities or the struggle to meet physicians in the facilities. Thus, they prefer to directly visit nearby CDROs”* (DM2)

Most of the pharmacy professionals and two decision makers discussed that the cost of healthcare made it unaffordable for patients to pay for services such as consultation and examination (e.g., laboratory work up etc.), as well as transportation costs. As a result, patients opted to directly purchase antibiotics from CDROs without a prescription as it is a more cost-effective option. This drives the demand for non-prescribed antibiotics from CDROs.

*“Patients note that they cannot afford the cost associated with healthcare visits or expert consultations, so that they chose to obtain antibiotics easily from CDROs”* (P4)

*“Given that the majority of patients have low financial capacity, they are neither interested nor able to pay expenses for laboratory work-up or x-ray diagnostic examination, therefore, they purposefully avoid examinations that require additional cost rather they tend to directly purchase antibiotics from CDROs”* (DM 2)

The majority of pharmacy professionals also described that many patients prefer not to travel to distant healthcare facilities or wait for services. As a result, they opted to directly buy antibiotics from CDROs, as it is their most time-efficient option.

*“Patients do not want to waste their time going far to healthcare facilities or in waiting some time to get the service. As most of the peoples visiting our CDRO are farmers, they are not interested to spend their time for healthcare facility visit rather they prefer a quick fix so as to go back to work as soon as possible. Thus, they come straight to the CDRO to get treatment including antibiotics”* (P6)

### Absence of or a weak prescription-only antibiotic sale regulatory enforcement or service supervision

Most pharmacy professionals and all decision makers stated that a lack of or a less rigorous enforcement of the regulation is a major factor for the dispensing of antibiotics without a prescription. There is infrequent oversight and few penalties for providing unprescribed antibiotics. For example, one professional noted that the CDRO is only inspected once in a year. The professionals emphasised that the profit interests of the owners are reinforced by the lack of a functioning regulatory system that conducts regular inspection or random surveillance, including prescription audits.

Most decision makers highlighted that some regulatory officials who conduct physical inspections of CDROs may neglect their work responsibilities for personal benefits on some occasions. CDROs take advantage of this loophole in the regulatory inspection to circumvent laws that limit the sale of antibiotics to prescription-only.

*“The main issue is the weak law enforcement or supervision. Had the implementation of the regulation been strong, there would not have been a situation in which one CDRO sells antibiotics without prescription while the other prohibits”* (P9)

*“I can say there is no strong regulatory supervision. For instance, I have seen everyone getting ceftriaxone and amoxicillin without prescription from many CDROs”* (P13)

Most decision makers noted that enforcement of the law or regular pharmacy supervision is significantly hampered by the insufficient numbers of regulatory supervisors throughout Ethiopia. Enforcing the regulation of dispensing antibiotics by prescription-only requires a large number of officials throughout the country. They also pointed out that limited involvement of CDRO staff in the regulation development has made enforcement difficult as CDROs are less co-operative and proactive in following the law. Some decision makers also acknowledged that CDROs can obtain antibiotics illegally from unauthorised suppliers in neighbouring countries without detection by regulatory bodies, allowing them to avoid penalties during audits.

“*There is no strict supervision and strong law enforcement system. It would not be unusual to see someone who has no profession or experience at the CDRO counter doing the dispensing”* (DM 4)

### Patient related factors (demand pressure)

#### Previous experience with the diseases and successful treatments

Several pharmacy professionals discussed that patients tend to purchase antibiotics directly from CDROs based on their own past experience of improving after taking an antibiotic or hearing positive outcomes from relative or friends. Some professionals also noted that some patients believe they are very familiar with the current illness and treatment, and therefore seek out CDROs to buy the same antibiotics they have used in the past. A decision maker concurred with this perspective.

*“They sometimes note that they have previously used a certain antibiotic for certain condition and was effective so that they believe it will do the same if they can purchase it directly from CDRO and use it now again when they feel that the condition is similar with the previous one”* (P4)

*“The other main issue is peer influence on patients, if there were similar cases in the family or neighbours and got cured after taking certain antibiotics, they prefer to directly purchase similar antibiotics from CDROs rather than visiting healthcare facilities”* (DM2)

### High antibiotic seeking culture

The majority of pharmacy professionals observed that some patients have a strong attachment to antibiotics. They have reported that there have been many occasions that patients visit CDROs with the specific aim of obtaining antibiotics by describing their symptoms and naming the antibiotics they want. Pharmacy professionals emphasised that some patients would go to great lengths to obtain antibiotics and sometimes, they may engage in heated conversation with CDRO staff if their requests are denied. They also noted that patients prefer to get antibiotics from CDROs because they feel that doctors at healthcare facilities are less likely to prescribe them antibiotics.

A decision maker also noted that patients often opt to purchase non-prescribed antibiotics because they believe having the medication on hand will provide them a sense of security.

*“The community usually explain that antibiotics are the medicines that gives them cure to their condition. The conditions that they are experiencing and the antibiotic they request may be totally unrelated, but they just say that I will be cured when I take amoxicillin and insist to have it by any means. They may even say it is my money I can purchase anything I want. Fundamentally, the community has a high attachment to antibiotics”* (P3)

*“Patients describe that the smoothest way to obtain antibiotics that they look for is going to CDRO and purchasing it. Otherwise, they believe denial of getting antibiotics is high if they visit healthcare facilities, and they do not want to take that risk”* (P3)

### Lack of knowledge and inappropriate attitudes about antibiotics use/supply or the dispensing regulation

Many pharmacy professionals stated that patients’ misunderstanding and/or inappropriate perceptions about antibiotic use and antibiotic dispensing regulation influence their demand for unprescribed antibiotics. They emphasised patients’ misconceptions to the extent that some patients perceive antibiotics as other commodities, with instances of patients visiting CDROs to request only one or two tablets of antibiotics based on their financial capacity. Some also believe that antibiotics gained directly from CDROs are more effective than those obtained through a prescription.

The Pharmacy professionals also noted that only a small number of patients understand the difference between over-the-counter and prescription-only medications, and almost none are able to differentiate in rural communities.

It was also noted that it is challenging to deny dispensing antibiotics to patients and explain the reasoning, as they perceive it as a poor treatment. They are only concerned with quick service and are neither knowledgeable or interested to know about the antibiotic dispensing regulation. In rural communities, in particular, refusals are often not accepted.

*“Upon arrival of clients to the CDRO, if you mention that antibiotics are only be provided with prescription, they instantly perceive as if you are deliberately refusing and disregarding their basic right and take it seriously as a mistreatment” (P1)*

*“Patients have no awareness about the dispensing regulation, and not interested to know about it. Thus, they perceive as they can walk-in to CDROs and purchase any medicine they want including antibiotics as long as the CDROs are established to sale drugs”* (P12)

Most decision makers acknowledged that patients, particularly those in rural towns, have a limited knowledge about appropriate antibiotic use and regulations surrounding antibiotic supply. They believed that this was one of the main factors for the high demand from patients for non-prescribed antibiotics. Rural community members also tend to stock up antibiotics, taking advantage of every opportunity to obtain them, such as during their trips to centre of towns on market days.

*“There is awareness gap in the community. Particularly, when you go out of the capital city to rural areas of the country, there is poor understanding across the community regarding the negative impact of non-prescribed antibiotic use, which has led to an increase in patients’ demand or use of antibiotics”* (DM2)

### Proposed solutions to reduce the non-prescribed sale of antibiotics

**Fig. 2:**
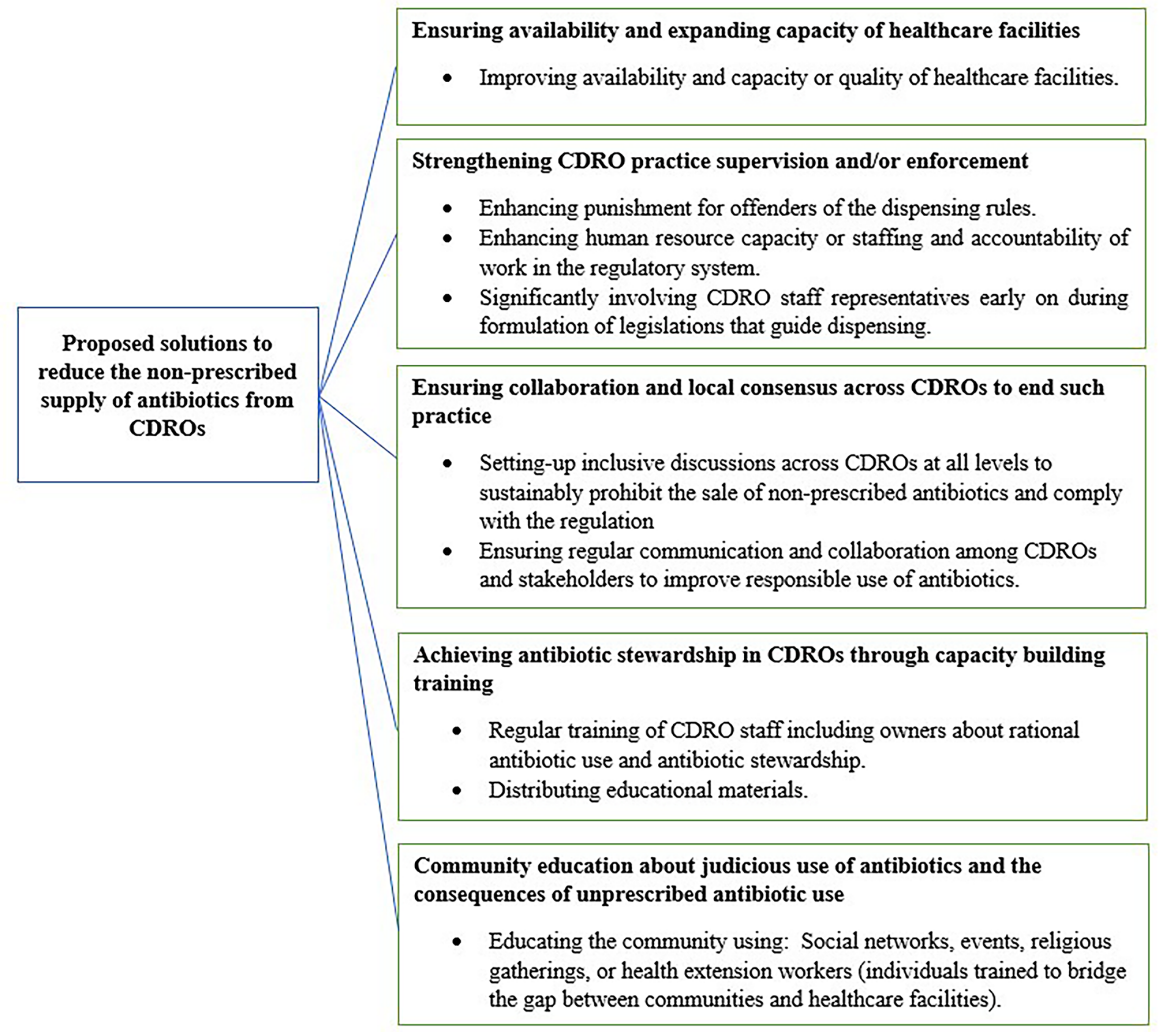
Proposed solutions to reduce the practice of dispensing antibiotics without prescription

The pharmacy professionals and decision makers suggested a suite of strategies to address the non-prescribed sale of antibiotics from CDROs including: (i) ensuring availability and expanding capacity of healthcare facilities, (ii) strengthening CDRO practice supervision and/or enforcement of laws, (iii) establishing collaboration and local consensus across CDROs to end such practice, (iv) achieving antibiotic stewardship in CDROs through capacity building training, and (v) educating the public about judicious use of antibiotics and the consequences of unprescribed antibiotic use (Fig. 2).

## Discussion

To our knowledge, this qualitative study is the first to examine the perspectives of multiple stakeholders on the drivers of, and solutions for, the problem of non-prescribed antibiotic dispensing by CDROs located in non-urban towns of Ethiopia. Thematic analysis of interviews with pharmacy professionals working in non-urban towns CDROs and high-level decision makers in the health system of Ethiopia revealed a shared set of factors that contribute to the non-prescribed sale of antibiotics, which can be grouped into categories of CDRO staff, health system, and patient related factors.

One major driver contributing to non-prescribed antibiotic dispensing is the profit-driven business model of CDROs and the financial difficulties of operating a CDRO in Ethiopia, which puts significant pressure on CDRO staff to increase sales.

This finding was supported by several similar studies conducted in LMICs [19, 34–37]. Previous systematic reviews of qualitative studies in LMICs also identified pharmacy professionals’ focus on financial gain as a key reason for dispensing antibiotics without a prescription [25, 26]. The burdensome and costly nature of opening a CDRO in Ethiopia is believed to lead CDROs to prioritise business goals to compensate for expenses such as rent and employees’ salaries, as well as associated loans. Government subsidies for CDROs, such as reducing the prices of medicines and rental fees, may help to alleviate this pressure.

The lack of knowledge or negative attitudes towards antibiotic dispensing among CDRO staff was also reported as a factor contributing to widespread non-prescribed antibiotic access, similar to other studies in LMICs [36, 38–40]. This could be partly because many of the actively working CDRO staff in Ethiopia, particularly in rural towns, are druggists with only a diploma in pharmacy, which may affect their understanding of appropriate antibiotic use, regulations, and the risks of ABR. Improving the knowledge and attitudes of CDRO staff would help to improve practices. However, it would not be sufficient to change dispensing practices alone, as their behaviour is also influenced by external factors such as pressure from owners or patients [25, 26].

Many professionals acknowledged that the lack or inadequacy of healthcare facilities leads to community members relying on CDROs as their primary source of treatment. This factor has been consistently reported in many studies across SSA [34, 37, 41, 42]. The reasons for this could be that the unavailability or unaffordability of healthcare facilities that leads people to rely on CDROs as their primary and sometimes only point of contact with the healthcare system. Many CDRO staff also admitted to being more lenient in providing antibiotics in these situations. To address this, implementing Ethiopia’s Health Sector Transformation Plan or Essential Health Service Package, which aims to achieve universal health coverage by improving healthcare infrastructure and trained health workforce, should be a priority [43, 44].

Another key driver reported was the lack of regular supervision or a lax enforcement of the prescription-only antibiotic dispensing policies. Several studies have shown the absence of practice supervision or ineffectiveness of law enforcement fuelling the non-prescribed antibiotic sale in CDROs [35, 36, 38, 41, 45]. Additionally, it is suggested that some regulatory delegates who conduct the physical inspection of CDROs may be dishonest in their work for personal benefits, and that there may be a lack of accountability in the legislative framework. In addition, inadequate human resource for regulation may result in an absence of or less frequent practice supervision throughout Ethiopia, consistent with a study conducted in Eritrea [41]. Stakeholders should discuss the obstacles to effective regulation and co-design strategies for improvement. Strict implementation of prescription-only policies should be a priority since other factors inducing the non-prescribed antibiotics sale are prominent because of the weak regulatory climate. This is in line with the strategic aims of Ethiopia’s Health Sector Transformation Plan and recent action plans for ABR prevention and containment [43, 44, 46].

Patient related factors or patient antibiotic demand pressure were also described to contribute to CDRO’s antibiotic dispensing practice. Factors such as lack of awareness or inappropriate attitudes in the community, self-medication based on previous experience, and a culture of high antibiotic seeking are described as contributing to this high demand. Studies in other LMICs have also shown that patient-related factors exacerbate the non-prescribed antibiotic sales [34–42, 45, 47, 48]. These factors suggest that community awareness campaigns or comprehensive educational interventions at the population level could be effective in addressing this issue and these measures have demonstrated effectiveness elsewhere [49]. Another strategy could be training CDRO staff to be antibiotic stewards, they are well-positioned to promote health and educate the community about appropriate antibiotic use. Changing community beliefs about antibiotics can help reduce their demand for antibiotics [49].

### Implication for policy and practice

The study identified several factors that contribute to the sale of non-prescribed antibiotics in rural CDROs, including individual factors related to patients and pharmacy professionals, organisational factors related to the business model of CDROs, and system-level factors related to healthcare facility accessibility and regulation/monitoring. To address these drivers, the study suggests ‘system-level’ reform that includes enforcing laws that govern, increasing the availability and capacity of primary healthcare facilities in rural areas, and reframing societal and pharmacy professional views of antibiotics and ABR through multi-faceted, multi-level interventions.

There are ways to address the issue of non-prescribed antibiotic sales in CDROs through regulatory or system-level measures. We suggest that the Ethiopian Food and Drug Authority increases resourcing and efforts in monitoring and enforcing policies related to non-prescribed antibiotic sales. Our finding emphasises the importance of involving CDROs and pharmacy professionals in designing, implementing, and evaluating policies related to non-prescribed antibiotic sales, so that they can take a more proactive role in addressing the issue. Additionally, the participants recommended rigorous inspections of CDROs, effective enforcement of laws through tough punitive mechanisms, and these measures reported to be effective in other countries [50–54]. In addition, increasing the active regulatory working staff is also important, as studies conducted in China and Eritrea have identified insufficient capacity to enforce existing legislation as a factor contributing to weak enforcement. These studies recommended efforts to improve the regulatory taskforce [41, 45]. Furthermore, ensuring good practice of the regulatory officials through enhanced accountability was also recommended. CDROs reported sourcing antibiotics from unauthorised suppliers illegally across borders and shutting down such illegitimate sources and regular prescription audits in the CDROs may help ensure effective tracking of the antibiotic transactions within CDROs.

Patients’ needs are a central value and prioritised by the CDRO staff, as highlighted in many previous studies [19, 34, 35, 45]. Addressing patients’ needs and increasing their knowledge about antibiotics through community education can help reduce demand for non-prescribed antibiotics in CDROs. It has been suggested that comprehensive and tailored community education through social events, religious gatherings, or health extension workers about the proper use of antibiotics and the dangers of non-prescribed antibiotic use may improve patients’ knowledge and reduce demand for antibiotics [49, 55, 56]. Additionally, the willingness of CDROs to provide non-prescribed antibiotics may be due to the staff’s inadequate knowledge or inappropriate attitudes about responsible antibiotic use and regulation. Continuing education or regular training or workshops for CDRO staff can improve their awareness or attitudes towards rational antibiotic provision or use and transform them into antibiotic stewards. This measure has been found to be effective in a previous study [57]. In addition to regulatory enforcements, setting up inclusive discussions among CDROs at all levels to sustainably refuse non-prescribed antibiotic sales and encouraging the government to subsidise CDROs with minimal business loans or lower the cost of important drugs as a way to improve rational use of antibiotics across CDROs.

### Strengths and limitations

This study is unique in that it examines both the perspectives of CDRO staff and high-level decision makers on the drivers contributing to and potential solutions for addressing non-prescribed antibiotic dispensing. Another strength of the study is that all invited participants agreed to participate and were willing to share their experiences and insights on the topic.

This study has some limitations. The qualitative study was primarily conducted to explain and give context to the reported ongoing non-prescribed sale of antibiotics in CDROs in the Amhara region of Ethiopia [18, 27]. Hence, we carried out the pharmacy professionals’ interviews in selected divisions of the Amhara region in Ethiopia and the findings may not be representative of other non-urban areas in the Amhara region or Ethiopia. Social desirability bias might have affected our findings, although the use of experienced interviewers with good probing skills likely minimised this bias. In addition, some factors such as the patient-related factors were reported from the perspectives of pharmacy professionals rather than patients. We also recognise the limitations associated with translating all the transcripts to English and conducting the initial line-by-line coding and generation of preliminary themes with only one researcher (SAB). Nevertheless, to ensure accuracy, the researcher engaged in frequent discussions with other team members (LH, LAS). Despite these limitations, this study adds to the existing limited evidence on this issue and its findings may serve as a foundation for developing interventions to prevent inappropriate use of antibiotics and promote antibiotic stewardship in CDROs.

## Conclusions

This study identified a variety of modifiable factors related to patients, CDRO staff, and legislation or health system that trigger the non-prescribed supply of antibiotics from CDROs with a focus on non-urban towns in Ethiopia. These include the business orientation of CDROs, patient demand pressures, and weak regulatory supervision or enforcement. These complex factors make it unlikely that a single intervention will be sufficient to address the issue. Instead, a range of multifaceted, integrated solutions have been proposed, including strengthening the legislative system, expanding healthcare services in the pursuit of universal health coverage, collaboration with and commitment by CDROs to end non-prescribed antibiotic sales, regular training for CDRO staff on the consequences of antibiotic misuse, and re-purposing social events to educate the community about rational use of antibiotics.

### Electronic supplementary material

Below is the link to the electronic supplementary material.

Additional file 1: Interview guide

## Data Availability

All relevant data are included in the paper.

## Declarations

### Reflexivity notes

The authors of this study are researchers with backgrounds in clinical pharmacy (SAB), health service research with expertise in epidemiology (LH) and public health physician and an infectious diseases epidemiology (LAS) with extensive experience in conducting qualitative and quantitative research in various aspects of public health. The interviewers were also researchers with backgrounds in pharmacy in Ethiopia. This study is a part of SAB’s PhD project through the University of Queensland. The research team was aware of their perspectives throughout the data collection, transcription and translation, coding, and analysis process, and attempted to treat all data equally.

## Abbreviations

ABR: Antibiotic resistance
AMR: Antimicrobial resistance
CDROs: Community drug retail outlets
DM: Decision maker LMICs:Low- and middle-income countries
SSA: Sub-Saharan Africa
