# Supplementary material for: “Handing out non-prescribed antibiotics is storing up trouble for the next generation!” Unpacking multistakeholder views of drivers and potential solutions in Ethiopia
Source: BMC Health Serv Res. 2023 Aug 7;23:830. doi: 10.1186/s12913-023-09819-4 (PMC10405379; doi:10.1186/s12913-023-09819-4)
Supplement: Supplementary file 1 — Additional file 1: Interview guide [file 12913_2023_9819_MOESM1_ESM.docx]

**“Handing out non-prescribed antibiotics is storing up trouble for the next generation!” Unpacking multistakeholder views of drivers and potential solutions in Ethiopia**

Sewunet Admasu Belachew^1, 2*^, Lisa Hall^1^, Linda A Selvey^1^

^1^School of Public Health, The University of Queensland, 288 Herston Rd, Herston, Qld 4006, Australia

^2^School of Pharmacy, Faculty of Medicine and Health Sciences, University of Gondar, Ethiopia

***Corresponding author**

Sewunet Admasu Belachew

School of Public Health, The University of Queensland, 288 Herston Rd, Herston, Qld 4006, Australia

Email: s.admasubelachew@uq.edu.au

**Authors’ email address**

Linda A Selvey: l.selvey@uq.edu.au

Lisa Hall: l.hall3@uq.edu.au

**Additional file 1: Interview Guide**

**Interview guide for CDRO Pharmacy professionals’ interview**

**Part 1: General information**

- Could you please tell us;
- Your age?
- Where your work place or facility is?
- About your work status (***Prompts***: owner? employee?)
- About your educational status (***Prompts***: master’s degree or bachelor’s degree or diploma certificate; pharmacist or druggist?
- Type of CDRO you currently work (***Prompts:*** pharmacy, drug store or rural drug vendor?)
- About your work experiences? How long have you worked in CDRO?

**Part 2: Exploring the supply and drivers for dispensing antibiotics including non-prescription dispensing of antibiotics.**

- 1. How important are antibiotics for treating people who are sick in the area you work?

***Prompt***: What do you know about antimicrobials? Tell us about antibiotics as compared to antimicrobials?

- What do you think about supplying antibiotics from CDROs?
  1. Let’s talk about how and why some CDROs dispense antibiotics without a prescription:

***Prompt:*** What do you think about this practice? What might be the reasons/drivers?

***Prompt****:* When do you decide to dispense antibiotics without prescription?

***Prompt****:* How do you decide what type of antibiotic to dispense? Are there any that you think are preferable or more effective?

***Prompt***: Could you tell us the common conditions for which non-prescribed antibiotics are provided? What do you think about the supply of antibiotics for mild and sever/emergency conditions?

***Prompt*:** What do you think about the supply of antibiotics for adults and children?

**Prompt**: How might distance of CDROs from health facilities affect antibiotic dispensing practice? If you think that dispensing might be higher in CDROs further from health facilities, why would that be and what could be done to reduce non-prescribed supply in these CDROs?

- 1. In your view, what strategy/ies could change the existing practice or reduce/control the non-prescription supply of antibiotics from CDROs?

***Prompt*:**  Who do you think should be responsible for changing existing practice? What people? What organisation? What part of government?

**Part 3**: **Exploring attitudes about and opportunities and challenges of antibiotic dispensing according to the regulations.**

- **Now we are going to talk about antibiotic dispensing according to the regulations**
- What do you know about the antibiotic dispensing regulations in Ethiopia?
- How important is the antibiotic dispensing regulations?

***Prompt:*** Do you dispense antibiotic according to the regulations? How could dispensing of antibiotic according to the regulations improve antibiotic dispensing practice and use?

***Prompt:*** What are the benefits and disadvantages to your CDRO of dispensing antibiotics according to the regulations?

***Prompt:*** How acceptable is dispensing of antibiotic according to the regulations to your colleagues /other CDRO staff?

- Let’s talk about how individuals and groups can influence whether you dispense antibiotics according to the regulations in CDROs: What do you think about the influences of:

Clients?

Colleagues?

Your employer?

Government or local authorities?

- In your opinion, what are the existing or potential opportunities or supports/enablers related to dispensing antibiotics according to the regulations in the CDRO setting?

***Prompt:*** What resources need to be in place to support implementation and continuity of dispensing antibiotics according to the regulations in CDROs?

***Prompt*:** Tell us about the skills you have for dispensing antibiotics according to the regulations in the CDROs? Is there anything you think might help (e.g., training etc.)?

- In your opinion, what are the main barriers or challenges to antibiotic dispensing according to the regulations in CDROs?
- How confident are you that any barriers in relation to antibiotic dispensing according to the regulations can be addressed?
- What would you recommend, change, or add to address the barriers and ensure the dispensing of antibiotics according to the regulations?
- In conclusion, if you have any final thoughts or additions, please feel free to share them?

**Interview guide for decision makers’ interview**

- What do you think about the antibiotic dispensing practice in CDROs of Ethiopia? What do you think about the non-prescription dispensing of antibiotics? What might be the reasons?
- What are the existing polices or regulatory laws in relation to over-the-counter dispensing of antibiotics by CDROs? Tell us the challenges of implementing these regulatory laws or policy principles to control the non-prescribed sale of antibiotics from CDROs?
- What are any additional challenges in the non-urban CDROs?
- What do you think about implementing antibiotic stewardship at CDRO level
- ***Prompts***: antibiotic dispensing according to regulation?
- In your view, what are the opportunities and challenges to implementing antibiotic stewardship at CDRO level
- ***Prompts:*** antibiotic dispensing according to the regulations?
- What would you recommend, change, or add to address the barriers and support CDRO staff to be active antibiotic stewards?
- In conclusion, if you have any final thoughts or additions, please feel free to share them?
